# Optical Spectrum and Photochemistry of Si_2_O_2_^+^

**DOI:** 10.1021/acs.jpca.4c08749

**Published:** 2025-02-12

**Authors:** Taarna Studemund, Kai Pollow, Marko Förstel, Alexander A. Breier, Otto Dopfer

**Affiliations:** Institut für Optik und Atomare Physik, Technische Universität Berlin, Hardenbergstraße 36, 10623 Berlin, Germany

## Abstract

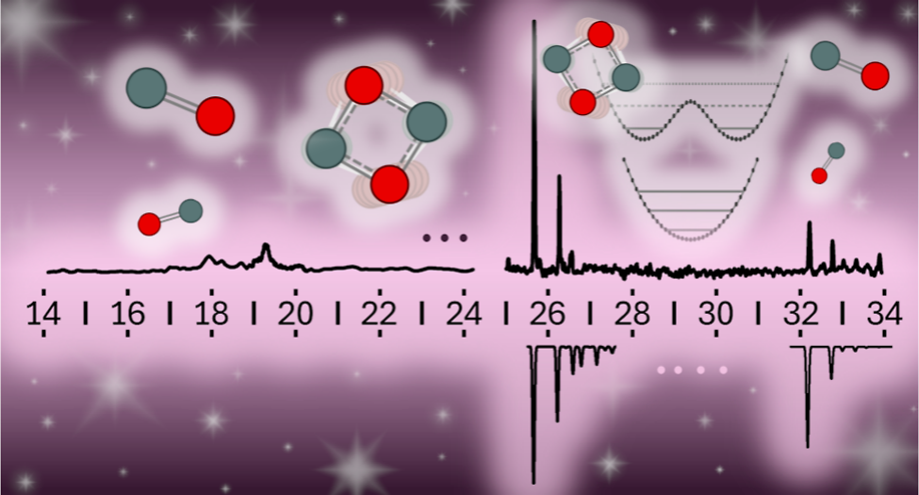

Silicates and silica are the major components of interstellar silicon-based dust grains and mainly composed of silicon and oxygen. Information about their geometric, electronic, optical, and photochemical properties is crucial for developing astrochemical models describing dust grain formation. To this end, we characterize herein the optical spectrum of mass-selected Si_2_O_2_^+^ cations in the 295–709 nm range using electronic photodissociation (EPD). The EPD spectra are recorded in a quadrupole/time-of-flight tandem mass spectrometer coupled to a laser vaporization source and compared to complementary time-dependent density functional theory (TD-DFT) calculations at the UB3LYP-D3/aug-cc-pVQZ level of theory, determining structures, energies, electronic spectra, and fragmentation energies of the low-energy isomers. The EPD spectrum is observed in the lowest-energy fragmentation channel, corresponding to SiO^+^ + SiO. The high calculated dissociation threshold of *D*_0_ = 4.60 eV (37,102 cm^–1^) requires two-photon absorption for EPD. The three electronic transitions observed at 19,264, 25,667, and 32,216 cm^–1^ are attributed to transitions from the doublet ground state of the most stable rhombic structure of Si_2_O_2_^+^ (*D*_2*h*_, ^2^B_1u_) into the first, fourth, and fifth excited doublet states, D_1_(^2^A_g_), D_4_(^2^B_2g_), and D_5_(^2^B_3u_), respectively. The resolved vibronic structure of the D_4_ and D_5_ state is analyzed by Franck–Condon Herzberg–Teller (FCHT) simulations to suggest vibrational assignments. The calculations indicate the reduction of symmetry from *D*_2*h*_ to *C*_2*v*_ in the D_4_ state along the ν_4_(b_1u_) coordinate (resulting in a flat double minimum potential), while the dipole-forbidden D_5_ state gains its vibronic intensity from HT coupling to the same ν_4_ mode.

## 1 Introduction

Silicon and oxygen are essential elements of our Earth’s crust and key components of, for example, sand and rocks. The analysis of interplanetary dust grains, meteorites, and other celestial objects from space revealed silicon compounds, mostly containing silica (SiO_2_) and metal silicates (e.g., M_2_SiO_4_ and MSiO_3_ with M = Fe or Mg). Thus, it is evident that silicon, oxygen, and their compounds (such as Si_*n*_O_*m*_) are crucial in the formation of Earth, planets, and other astronomical bodies.^1−8^ The first stable Si_*n*_O_*m*_ compound identified in space, SiO, was detected by radioastronomy already in 1971.^9−11^ Beyond this diatomic, no further (polyatomic) Si_*n*_O_*m*_ molecule has been detected in the interstellar medium (ISM).^12−14^ On the other hand, broad and nonspecific infrared (IR) bands around 10 and 18 μm observed in the spectra of stars, meteorites, and interstellar dust grains have been assigned to Si–O stretching and bending modes of silicate grains.^3,5,6,15−19^ Furthermore, silicates are considered potential sources of the strong discrete interstellar excitation features at 217.5 nm in the UV range^20,21^ and the broad extended red emission (ERE) in the VIS to NIR range.^22,23^ The link between these two size regimes, from diatomic SiO to large μm-sized dust particles in the ISM, remains a long-standing problem in astrochemistry. A variety of recent theories consider grain formation via a bottom-up approach in the gas phase followed by further particle growth at the surface.^2,3,24−26^ However, details of the particle growth mechanism are still open questions, including the core formation, the structure and reactivity of intermediates, the chemical composition, and size distribution of the grains, and the role of temperature and the radiation field.^3,27−29^ Indeed, several laboratory experiments demonstrate that SiO can aggregate and form larger particles even at low temperature conditions, indicating that bottom-up aggregation involving low or vanishing barriers is indeed feasible under astrophysical conditions.^30−34^ The growth of such Si_*n*_O_*m*_ clusters has been simulated using quantum chemical methods for both stoichiometric (*n* = *m*) and non-stoichiometric (*n* ≠ *m*) cases^3,20,35−37^ and experimentally investigated by photoionization mass spectrometry.^20,36^ Additional simulations include the growth of metal silicates.^3,29,38,39^

Besides neutral silica and silicate particle growth, ion–molecule reactions may also significantly contribute to silicon chemistry in specific astrophysical environments, as they have larger cross sections in low-density media.^40−45^ Due to their high reactivity and low density, the spectroscopic characterization of ions is challenging.^46^ Consequently, the present information on the geometrical and electronic structure, chemical bonding and reactivity, and photochemistry of polyatomic Si_*n*_O_*m*_^+^ ions mainly relies on computational chemistry and mass spectrometry experiments.^47−57^ Spectroscopic data required for the experimental characterization of the geometric and electronic structure are rather sparse. Regarding Si_*n*_O_*m*_^+^ cations, SiO^+^ is well characterized by calculations and vibronic spectroscopy.^58−64^ The structures of stoichiometric (SiO)_*n*_^+^ clusters with *n* = 3–5 have been derived from multiple photon dissociation (IRMPD) spectra observed in the SiO loss channel and assigned to the most stable cyclic (*n* = 3) and bicyclic (*n* = 4–5) isomers through comparison to density functional theory (DFT) calculations at the B3LYP/cc-pVTZ level.^65^ In addition, the structures of a larger variety of colder Xe-tagged Si_*n*_O_*m*_^+^ ions with *n* = 3–5 and *m* = *n* and *n* ± 1 have been derived from IRMPD.^66^ The assigned lowest-energy structures indicate several recurrent bonding motifs, including the Si_2_O_2_ rhombus, the Si_3_O_2_ pentagon, and the Si_3_O_3_ hexagon. The rather stable tetrahedral SiO_4_ unit typical for silicates appears in essentially all assigned Si_*n*_O_*m*_^+^ structures with *m* ≥ 4, providing compelling experimental evidence that interstellar silicates may indeed grow via a bottom-up approach starting from small silicon-oxide-based molecules, clusters, and ions. Until recently, no electronic spectra have been available for any polyatomic Si_*n*_O_*m*_^+^ and Si_*n*_C_*m*_^+^ cations (as well as neutral Si_*n*_O_*m*_ clusters). Such spectra are however required to determine the optical and photochemical properties. To this end, we have started a research effort to systematically investigate the optical spectra of mass-selected Si_*n*_O_*m*_^+^ and Si_*n*_C_*m*_^+^ ions by electronic photodissociation (EPD) in a tandem mass spectrometer coupled to a temperature-controlled laser vaporization source. The developed setup has initially been validated and optimized by measuring EPD spectra of a variety of Au-containing clusters.^67−72^ Subsequently, EPD spectra have been obtained for a variety of fundamental Si-containing ions, including Si_4_C_2_^+^ as a first example for a Si_*n*_C_*m*_^+^ ion,^73^ diatomic Si_2_^+^,^74^ the Si_3_O_2_^+^ pentagon,^13^ and the mixed triatomic SiO_2_^+^ and Si_2_O^+^ ions,^80^ providing the first experimental information about their optical and photochemical properties by comparison to time-dependent DFT (TD-DFT) calculations. Herein, we extend this series to the rhombic Si_2_O_2_^+^ ion by reporting three well-separated electronic transitions in the visible to UV range. We compare these observed and previously unknown electronic states with TD-DFT calculations to identify them and thus obtain essential properties of this potential dust grain precursor relevant for astrochemical studies.

Previous matrix IR studies have shown that neutral Si_2_O_2_, a fundamental building block for larger silicon oxide structures, has a planar rhombic structure with *D*_2*h*_ symmetry,^75^ in line with sophisticated computational results.^76−78^ Mass spectrometric and computational studies suggest that this structure is also the most stable isomer for the cation.^53,56,66,77^ Upon ionization (ionization energy IE = 9.2 eV), Si_2_O_2_^+^ maintains a planar rhombic structure (*D*_2*h*_ symmetry) with slightly shorter Si–O bonds (1.70 Å) compared to the neutral (1.71 Å).^56,76,77^ Computational studies suggest SiO loss as the lowest energy fragmentation channel.^56,76^ Three IR active bands are predicted, with the strongest at 800 cm^–1^ accompanied by weaker bands at ∼300 and ∼520 cm^–1^ as extracted from Figure 3 in ref (56). Further, two Raman bands are computed at 860 and 574 cm^–1^.^56^ Because the structures of the neutral and the cation are rather similar, and further, the neutral Si_2_O_2_ rhombus is considered a promising building block for larger silicon oxide aggregates, detailed studies on the Si_2_O_2_^+^ cation are also of interest.^76^

## 2 Experimental Setup and Computational Methods

EPD spectra of Si_2_O_2_^+^ are recorded in the range of 1.75–4.21 eV (14,104–33,922.8 cm^–1^, 294.8–709.4 nm) using a quadrupole/time-of-flight tandem mass spectrometer described in detail elsewhere.^71,79^ The setup has been used before for recording vibronic EPD spectra of a variety of gold cluster ions^67−72^ as well as Si-containing cluster ions such as Si_2_^+^, Si_4_C_2_^+^, SiO_2_^+^, Si_2_O^+^, and Si_3_O_2_^+^.^13,73,74,80^ Briefly, Si_*n*_O_*m*_^+^ clusters are generated in a plasma produced in a laser vaporization source using a pulsed Q-switched Nd:YAG laser (532 nm, 2–10 mJ, 20 Hz, diameter 0.5 mm) focused onto a rotating and translating Si rod (American Elements 99.9%) using a lens with *f* = 28 cm. The plasma is seeded in a rare gas mixture (8 bar) containing O_2_ reaction gas (0.05% O_2_/He or 0.1% O_2_/Ar). Similar to previous experience, the Ar gas mixture produces colder clusters and thus EPD spectra with narrower peaks and better signal-to-noise ratio.^13,74,80^ Hence, we report only the analysis obtained using this gas mixture. The cluster ions pass through a reaction channel with a temperature-controlled nozzle with a diameter of 1 mm held at 300 K before expanding into vacuum. Cooling of the nozzle resulted in much lower parent ion signal and is thus not employed. After the generated Si_*n*_O_*m*_^+^ clusters pass through the skimmer, a quadrupole mass filter selects the desired Si_2_O_2_^+^ ions (*m*/*z* 88). These are guided through an Einzel lens system into the extraction region of an orthogonal reflectron time-of-flight mass spectrometer (ReTOF-MS), where resonant EPD is induced by a laser pulse (2–3 mJ) emitted from a tunable optical parametric oscillator (OPO, 5–10 cm^–1^ bandwidth, 192–2750 nm tuning range, 0.5–150 mJ/pulse, 10 Hz, diameter 2.5–3 mm) pumped by a Q-switched Nd:YAG laser (355 nm, 290 mJ). The OPO laser pulse is fired 0.4–0.5 μs before ion extraction into the ReTOF-MS. At the end of the ReTOF-MS, both the generated daughter ions and the remaining parent ions are detected using a dual-stage microchannel plate detector. The fragmentation laser operates at 10 Hz (on signal), while clusters are generated at 20 Hz. Finally, the EPD signal is analyzed using the parent and daughter ions of the laser-on signal traces averaged over 200 shots for each wavelength and normalized for OPO laser flux. Only the monoisotopic species are considered for the EPD analysis. Initial overview spectra are acquired in the range 295–709 nm using a step size of 1 nm. Spectral ranges with resonant absorption bands are then measured at a reduced step size (0.01 nm for 389.0–390.5 nm, 0.1 nm for 294.8–400.0 nm). Due to insufficient OPO laser power, the range 400–410 nm is excluded. The accuracy of the experimentally derived band centers is assumed to be 10 cm^–1^, although the observed broad band intensity distribution resulting from partially unresolved (ro-)vibrational hot bands is thermally affected by the population distribution of the ground state.

DFT calculations coupled to a basin-hopping algorithm are employed to locate the lowest-energy isomers of Si_2_O_2_^+^.^81^ Based on a previously described algorithm at the RI-BP86/def-SVP level,^82,83^ as implemented in the TURBOMOLE V6.3 program package,^84^ we identify a variety of nonequivalent structures. The five lowest-energy isomers are then further optimized at the UB3LYP/aug-cc-pVQZ level of theory including the Grimme dispersion correction with Becke-Johnson damping (GD3BJ)^85,86^ using the GAUSSIAN16 program package.^87^ Excited state calculations are conducted at the TD-DFT level using the same theoretical level. For comparison, (TD-)DFT calculations are also performed at the UCAM-B3LYP/cc-pVTZ level, which yields results similar to the UB3LYP data. We note that energies of various Si_*n*_O_*m*_ clusters derived at the computationally more-demanding MP2 level have also been reported to be similar to those at the B3LYP level.^76^ In general, the quartet states of all five isomers are calculated to be higher in energy than the corresponding doublet states (Δ*E* = 3.788, 3.209, 0.002, 1.297, 0.509 eV for **I–V**), which agrees with previous studies for isomer **I**.^53,66,77^ Only for **III**, the doublet and quartet states are similar in energy. However, this is not relevant for the present study, because **III** is rather high in energy compared to **I** (Δ*E* > 4 eV) and thus not expected in the experiment. The few computational studies available for neutral^56,75−77,88−90^ and cationic^56,76,77^ Si_2_O_2_^(+)^ agree with our results regarding the planar rhombic ground state geometry with *D*_2*h*_ symmetry. For all stationary points on the potential, harmonic vibrational frequencies are determined to confirm their character as minima or transition states and to derive relative energies (*E*_0_) and dissociation energies (*D*_0_) corrected for vibrational zero-point energy. Vertical TD-DFT spectra computed at the UB3LYP/aug-cc-pVQZ level yield excitation energies and oscillator strengths for the first seven excited states of each isomer. Franck–Condon simulations with Herzberg–Teller coupling (FCHT), as implemented in GAUSSIAN16, are used in combination with PGOPHER^91^ to assign the resolved vibronic structure. In general, initial simulations are conducted for *T* = 1 K to avoid hot bands and to focus on the assignment of the excited state vibrations. Subsequent simulations conducted at higher *T* include hot bands. Charge distributions are analyzed by natural bond orbital (NBO) analysis, while natural transition orbitals (NTO) are used to visualize the orbitals involved in each excitation.

## 3 Results and Discussion

**Figure 1:**
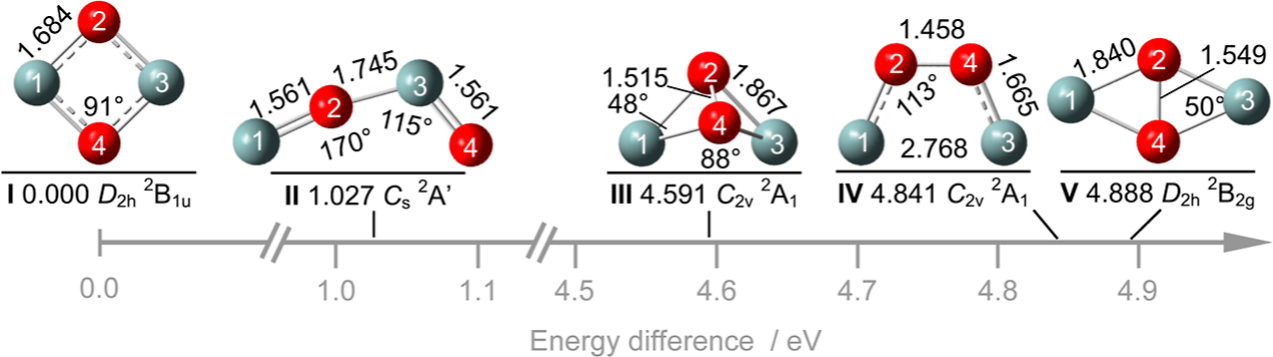
Minimum structures of the five most stable Si_2_O_2_^+^ isomers (**I**–**V**, O in red, Si in gray) obtained at the UB3LYP/aug-cc-pVQZ level, along with bond lengths and selected bond angles (in Å and degrees), relative energies (*E*_0_ in eV), structural and electronic symmetries.

The five most stable isomers of Si_2_O_2_^+^ (**I–V**) obtained at the UB3LYP/aug-cc-pVQZ level are shown in Figure 1, along with relevant geometric parameters, relative energies, and symmetries of the electronic ground states.

Isomer **I** has a planar rhombic structure with *D*_2*h*_ symmetry and a ^2^B_1u_ electronic ground state with a ···(a_g_)^2^(b_3g_)^2^(b_3u_)^2^(b_1g_)^2^(b_2u_)^2^(a_g_)^2^(b_1u_)^1^ electronic configuration. The Si–O bond length of 1.684 Å is typical for a bond order of 1.5 (average 1.623 ± 0.100 Å).^92^ The distance between the two Si atoms of 2.409 Å is not much longer than the average known bond lengths of neutral Si–Si bonds (average 2.331 ± 0.100 Å).^92^ The distance between the two O atoms (2.353 Å) is comparable to the Si–Si distance but much longer than the average neutral O–O bond length (1.246 ± 0.300 Å)^92^ suggesting the lack of a chemical O–O bond in **I**, consistent with O to bind to at most to two atoms. In the almost square structure, the Si–O–Si angle is slightly larger than the O–Si–O angle (91° > 89°). Comparison between the neutral and the cationic isomer **I** reveals the impact of ionization on structural and electronic properties. The neutral molecule has also a planar rhombic structure with *D*_2*h*_ symmetry in its ^1^A_g_ electronic state. Removal of an electron from the nonbonding b_1u_ orbital localized mostly on the Si atoms causes almost no change in the geometric square structure (Δ*r*_SiO_ = −0.016 Å, Δβ_SiOSi_ = −1.5°, Δβ_OSiO_ = +1.5°) and requires an ionization energy of 9.111 eV. The atomic charge distribution for the neutral (*q*_Si_ = 1.349 and *q*_O_ = −1.349 *e*) and the cation (*q*_Si_ = 1.752 and *q*_O_ = −1.252 *e*) indicates that the electron is removed from the lone pair orbitals of the much less electronegative Si atoms. Our calculated IR active modes agree with those of previous calculations.^56^

While isomer **I** has a symmetric cyclic structure, the next isomer **II** in energy (Δ*E*_0_ = 1.027 eV) has a planar chain-like structure with β_OSiO_ = 115° in its ^2^A’ ground state. Despite its high energy, it may be formed in the hot plasma of the laser vaporization source. However, its relatively low barrier for ring closure toward **I** (0.103 eV) suggests possible conversion from **II** to **I** in the hot plasma. Isomers **III–V** all have a O–O bond (*r*_OO_ ≈ 1.5 Å) and are much higher in energy (*E*_0_ = 4–5 eV). Hence, they are not expected to be populated in the expansion. Isomer **III** has a nonplanar *C*_2*v*_ structure in its ^2^A_1_ state, while **IV** has a planar open-ring *C*_2*v*_ structure with only two Si–O bonds in the Si–O–O–Si arrangement of its ^2^A_1_ state. Similar to **I**, **V** has a planar rhombic *D*_2*h*_ structure in its ^2^B_2g_ state but is strongly elongated along the Si–Si axis and thus features a short O–O bond. As mentioned above, the used ion source typically generates the most stable isomer of a given ion.^13,66,74,79,93^ Hence, we focus our discussion below mostly on the excited states of isomer **I**.

The dissociation energies (*D*_0_) of isomer **I** of Si_2_O_2_^+^ calculated for all possible two-body fragmentation processes are listed in Table 1. Only dissociation into two fragments is considered, as three-body fragmentation is usually more energy-demanding, as it involves breaking of more chemical bonds. The lowest fragmentation threshold is into SiO^+^ and SiO with *D*_0_ = 4.60 eV. This channel is expected to be relatively low in energy, because of the rather strong bonds of both neutral and cationic SiO (*D*_0_ = 7.93 and 4.5 eV).^94^ The next channel into Si^+^ + SiO_2_ is also relatively low in energy (*D*_0_ = 5.01 eV) due to the low ionization energy of Si (8.15 eV^95^) and the high stability of SiO_2_ (3.2 eV).^96^ The third decay channel is fragmentation into Si_2_O^+^ + O and requires *D*_0_ = 5.82 eV. All other channels require more than 8.5 eV for fragmentation. Interestingly, even the lowest fragmentation pathway calculated at *D*_0_ = 4.60 eV is above the investigated spectral range (1.75–4.21 eV). Thus, according to our calculations, EPD signal originating from the ground electronic state of **I** can only be generated by the absorption of two or more photons.

**Table 1:** Dissociation Energies (*D*_0_ in eV, UB3LYP/aug-cc-pVQZ) of all Two-Body Fragmentation Channels of Isomer **I** of Si_2_O_2_^+^^a^

fragments		UB3LYP
**SiO**^**+**^ **+ SiO**	**(**^**2**^**Σ**^+^ **+** ^**1**^**Σ**^**+**^**)**	**4.60**
Si^+^ + SiO_2_	(^2^*P*_1/2_ + ^1^A_1_)	5.01
Si_2_O^+^ + O	(^2^Π_g_ + ^3^P_2_)	5.82
Si_2_^+^ + O_2_	(^4^Σ_g_^–^ + ^3^Σ_g_^–^)	8.56
SiO_2_^+^ + Si	(^2^A_2_ + ^3^P_0_)	9.28
O^+^ + Si_2_O	(^4^S_3/2_ + ^3^Σ_g_)	12.97
O_2_^+^ + Si_2_	(^2^Π_g_ + ^3^Σ_g_^–^)	13.24

a The most stable structure with lowest-energy electronic and spin configuration is used for each fragment. The observed fragment pathway is indicated in bold.

The vertical excitation energy (*E*_v_) and oscillator strength (*f*) of the transitions predicted from the ground state of isomer **I** into its first seven excited states by TD-DFT are listed in Table 2. The energies range from 2.57 to 5.18 eV. According to the dipole selection rules for molecules with *D*_2*h*_ symmetry, only states with A_g_, B_2g_, and B_3g_ symmetry are allowed from the B_1u_ ground state. To this end, only the D_1_(^2^A_g_) and D_4_(^2^B_2g_) states at 2.565 and 3.528 eV (20,688 and 28,455 cm^–1^) have nonvanishing oscillator strengths of *f* = 0.1542 and 0.0198, whereby these differ by almost 1 order of magnitude. The transitions predicted for isomer **II** have all very low oscillator strengths (*f* < 0.006), with the exception of D_6_ (*f* = 0.0181, *E*_v_ = 3.68 eV or 29,682 cm^–1^). Isomer **III** has more than one significant transition, with *f* > 0.01 for D_3_ (*f* = 0.0565, *E*_v_ = 3.30 eV or 26,610 cm^–1^) and D_5_ (*f* = 0.1327, *E*_v_ = 3.87 eV or 31,230 cm^–1^). Most transitions of isomer **IV** have *f* ≤ 0.005, with the exception of D_4_ (*f* = 0.0367, *E*_v_ = 3.27 eV or 26,364 cm^–1^) and D_2_ (*f* = 0.0129, *E*_v_ = 1.93 eV or 15,593 cm^–1^). All transitions of isomer **V** in the scanned spectra range have *f* ≤ 0.0006. Overall, the spectrum predicted for the most stable isomer **I** is very different from all other calculated spectra and exhibits only a few transitions (vide infra). In particular, its first transition into the D_1_ state (*E*_v_ = 2.57 eV or 20,688 cm^–1^) is predicted to be its strongest one and also quite close to half of its dissociation energy (*D*_0_/2 = 2.30 eV or 18,551 cm^–1^).

**Table 2:** Vertical Excitation Energy (*E*_v_) and Oscillator Strength (*f*) of the Excited States (D_*n*_) of Isomer **I** of Si_2_O_2_^+^ (*D*_2*h*_) Calculated at the UB3LYP/aug-cc-pVQZ Level^a^

state	*E*_v_/eV (cm^–1^)	*f*
D_0_(^2^B_1u_)	0 (0)	
D_1_(^2^A_g_)	2.565 (20,688)	0.1542
D_2_(^2^B_2u_)	2.626 (21,180)	0.0000
D_3_(^2^B_1g_)	2.811 (22,672)	0.0000
**D**_**4**_**(**^**2**^**B**_**2g**_**)**^b^	**3.528 (28,455)**	**0.0198**
**D**_**5**_**(**^**2**^**B**_**3u**_**)**	**3.789 (30,560)**	**0.0000**
D_6_(^2^B_3u_)	4.890 (39,440)	0.0000
D_7_(^2^B_3u_)	5.178 (41,763)	0.0000

a The D_4_ and D_5_ states assigned to bands **B** and **C** in the EPD spectrum are indicated in bold.

b Optimization of D_4_ leads to a ^2^B_1_ state of a structure with *C*_2*v*_ symmetry.

**Figure 2:**
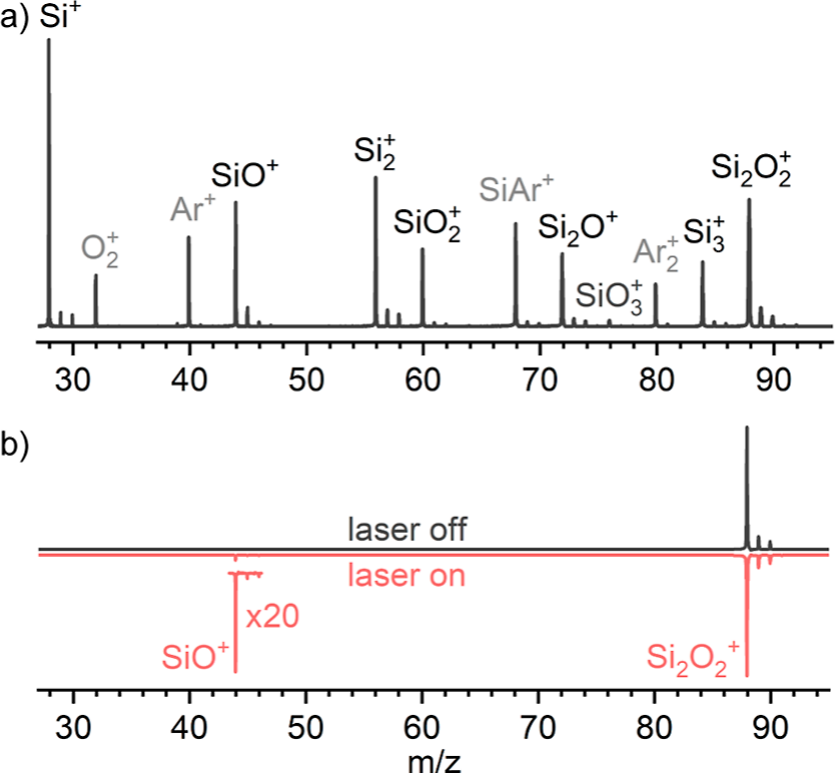
(a) Typical mass spectrum of Si_*n*_O_*m*_^+^ ions produced by laser vaporization of a Si rod and seeding the resulting plasma in a O_2_/Ar gas mixture, along with assignments. (b) EPD mass spectra of mass-selected Si_2_O_2_^+^ ions (*m*/*z* 88–90) with OPO laser off (black) and laser on (red, λ = 522.6–526.5 nm, band **A**), demonstrating the exclusive generation of the SiO^+^ fragment ion.

A typical mass spectrum of the laser vaporization ion source for conditions optimizing the production of Si_2_O_2_^+^ (Figure 2a) reveals a variety of Si_*n*_O_*m*_^+^ ions in the mass range *m*/*z* 27–95 (e.g., Si_*n*_^+^, Si_*n*_O^+^, Si_*n*_O_2_^+^, O_2_^+^), along with ions arising from the Ar carrier gas (e.g., Ar_*n*_^+^, SiAr^+^). The isotope distribution measured for Si_2_O_2_^+^ (*m*/*z* 88–90) compares favorably with the one simulated using natural abundances of the ^28–30^Si isotopes (82(3):12(3):6(3) versus 85:9:6), indicating that the Si_2_O_2_^+^ mass peaks are not contaminated by other isobaric ions.

**Figure 3:**
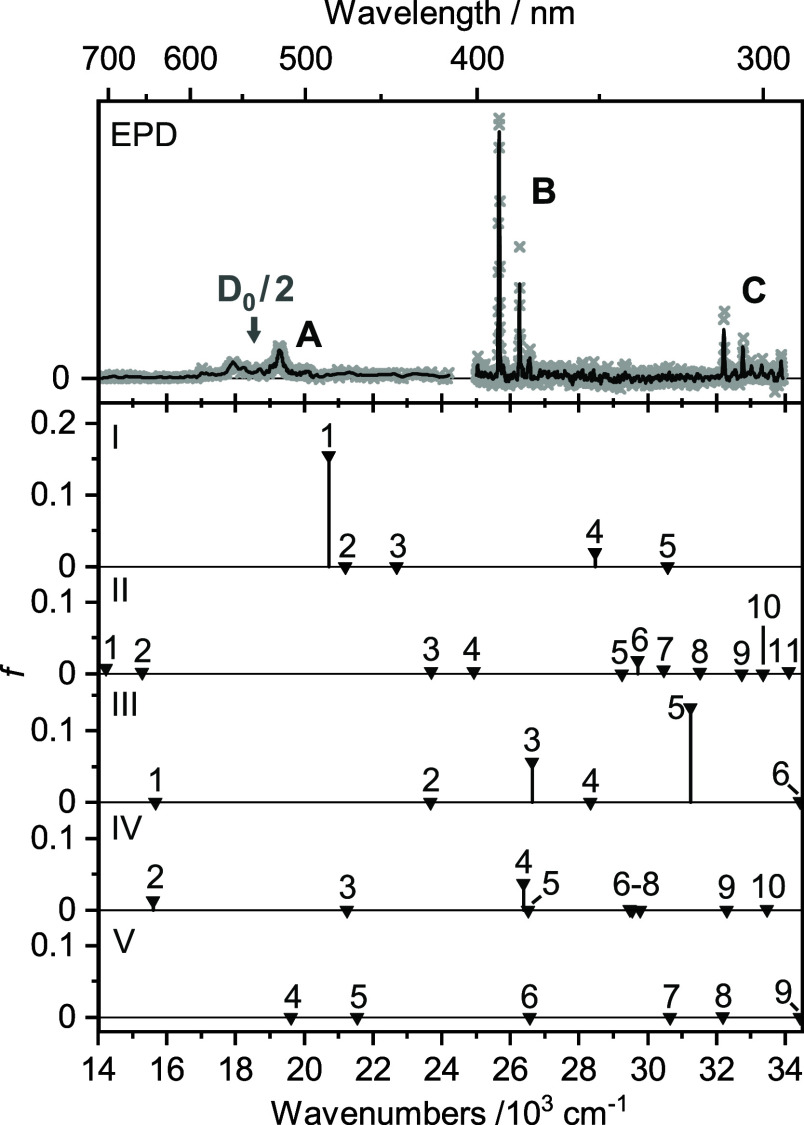
Comparison of the EPD spectrum of Si_2_O_2_^+^ obtained in the SiO^+^ fragmentation channel (linear scale) using the O_2_/Ar buffer gas mixture compared to vertical absorption spectra (oscillator strength *f*) computed for the five most stable isomers (**I–V**) at the UB3LYP/aug-cc-pVQZ level. The gray crosses in the EPD spectrum correspond to the individual data points, while the solid black line is generated by a moving average filter (Loess, 11 points). The arrow indicates the position of half of the dissociation energy of isomer **I** calculated as *D*_0_/2 = 2.30 eV (18,551 cm^–1^). The 24,390–25,000 gap is excluded from the EPD spectrum because of insufficient intensity of the OPO laser in the range 400–410 nm.

For initial EPD mass spectra (Figure 2b), the three main isotopologues of Si_2_O_2_^+^ (*m*/*z* 88–90) are mass-selected with the quadrupole and irradiated with the OPO laser scanned in the range 522.6–526.5 nm (band **A**, Figure 3). The mass spectra are shown for laser off (black) and on (red). In agreement with the predictions of the lowest-energy dissociation channel, SiO^+^ is the only fragment observed. This is also true for the other spectral ranges covered in this work. The fact that SiO^+^ is observed in the 525 nm range (2.36 eV) implies that at least two photons have to be absorbed to overcome the dissociation threshold computed as 4.6 eV. Analysis of the fragment ion signal as a function of OPO laser intensity yields a linear dependence suggesting a resonant 1 + 1 process. The observed fragment yield of (up to) 2.5% for the relatively weak band **A** indicates a good overlap between ion and laser beams.

The full wavelength-dependent EPD spectrum obtained for mass-selected Si_2_O_2_^+^ (*m*/*z* 88) in the range 14,104–33,923 cm^–1^ (Figure 3) exhibits a weaker broader feature **A** peaking at 19,264 cm^–1^ and two main bands **B** and **C** with band origins at 25,667 and 32,216 cm^–1^ associated with well-resolved vibronic structure. In an effort to assign these bands to electronic states of specific isomers, the EPD spectrum of Si_2_O_2_^+^ is compared in Figure 3 to vertical excitation spectra of the isomers **I–V**. Initial inspection of the position and intensities of the predicted transitions suggests an assignment of the bands **A–C** to the D_1_, D_4_, and D_5_ states of the most stable isomer **I**, when taking into account that band **C** assigned to the D_5_ state gains its intensity by Herzberg–Teller (HT) coupling not included in the vertical TD-DFT calculations. The agreement with the spectra predicted for the much less stable isomers **II–V** is much worse with respect to number, position, and relative intensity of the transitions. Hence, isomer **I** is concluded to be the sole carrier of the measured EPD spectrum.

Band system **A** occurs in the range 16,863–21,598 cm^–1^ (463–593 nm) and shows broader and partly resolved substructure. Its most intense feature occurs at 19,264 cm^–1^ with fwhm = 205 cm^–1^ and some weaker peaks at 20,084 and 21,322 cm^–1^ with fwhm = 276 and 514 cm^–1^. The band is assigned to the allowed D_1_(^2^A_g_) ← D_0_(^2^B_1u_) transition of isomer **I** predicted at 20,688 cm^–1^ (2.57 eV) with significant oscillator strength by the vertical TD-DFT calculations. Interestingly, the transition occurs close to one-half of the dissociation energy of **I** predicted as *D*_0_/2 = 2.30 eV or 18,551 cm^–1^. Hence, it appears close to the two-photon dissociation threshold, which may account for its weak relative intensity in the EPD spectrum when compared to the high computed oscillator strength. This coincidence may also account for the somewhat broader peaks observed in the EPD spectrum, because only hot bands may possibly be probed in such a two-photon process. Hence, we refrain from a more detailed vibronic analysis of band **A** and focus in more detail on the well-resolved bands **B** and **C**.

**Figure 4:**
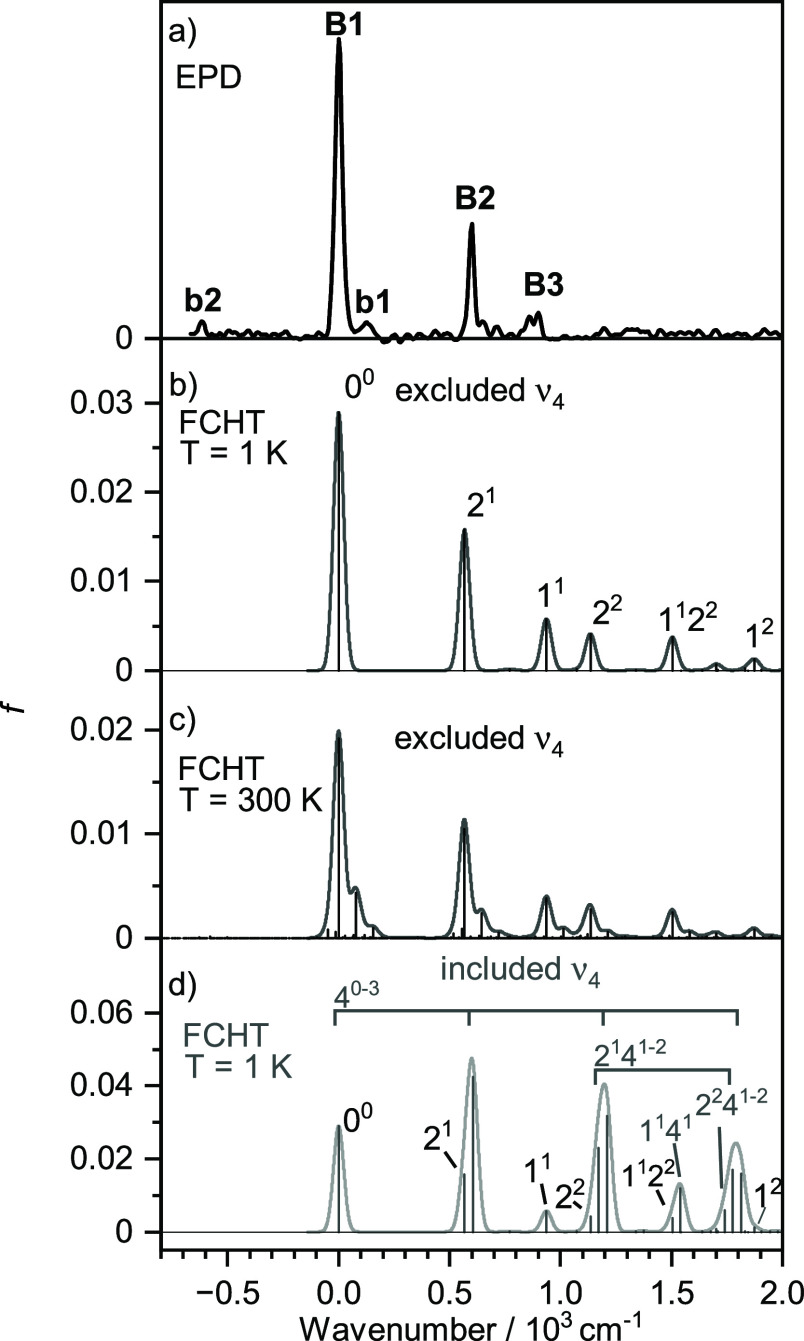
Comparison of band **B** of the experimental EPD spectrum of Si_2_O_2_^+^ (a, linear scale) to harmonic FCHT simulations (b–d, shifted by −27,624 cm^–1^) as a function of internal energy, along with vibrational assignments: simulation without ν_4_ at *T* = 1 K (b) and 300 K (c) and with ν_4_ at *T* = 1 K (d).

Band system **B** occurs in the range 25,580–26,610 cm^–1^ (375–390 nm) and consists of three main peaks labeled **B1–B3** and two much weaker features **b1** and **b2**, as shown in the expanded view in Figure 4. Peak **B1** located at 25,667 cm^–1^ (fwhm 40 cm^–1^) is assigned to the D_4_(^2^B_2g_) ← D_0_(^2^B_1u_) transition computed at 28,455 cm^–1^ by the vertical TD-DFT calculations. Vibronic bands **B2** and the doublet **B3** in the D_4_ state are centered at 26,267 and 26,567 cm^–1^ (fwhm 34 and 81 cm^–1^), giving rise to vibrational frequencies of 600 and 900 cm^–1^, respectively. The width of the bands may arise from a combination of the limited laser bandwidth, unresolved rotational substructure, (sequence) hot bands, and lifetime broadening. The weak features **b1** and **b2** at 25,050 and 25,793 cm^–1^ (fwhm 59 and 96 cm^–1^) occur at −617 and +126 cm^–1^ with respect to the **B1** origin band.

**Figure 5:**
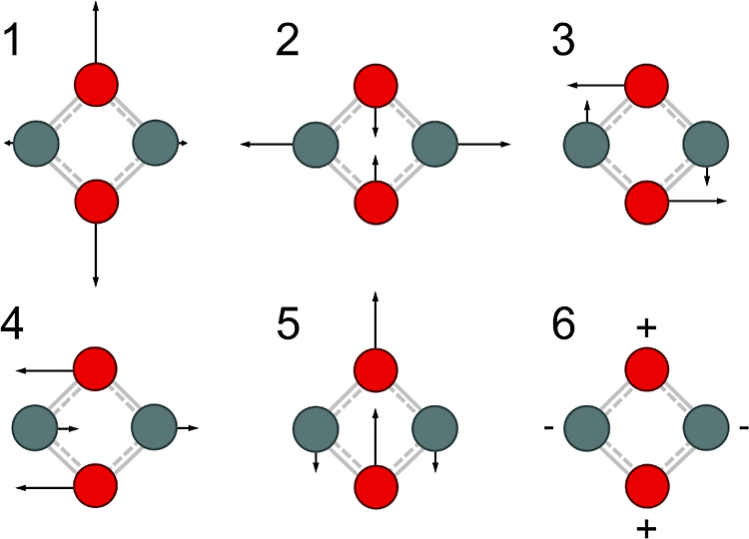
Visualization of the normal modes ν_1_–ν_6_ of isomer **I** in the D_0_ state (Table 3).

**Figure 6:**
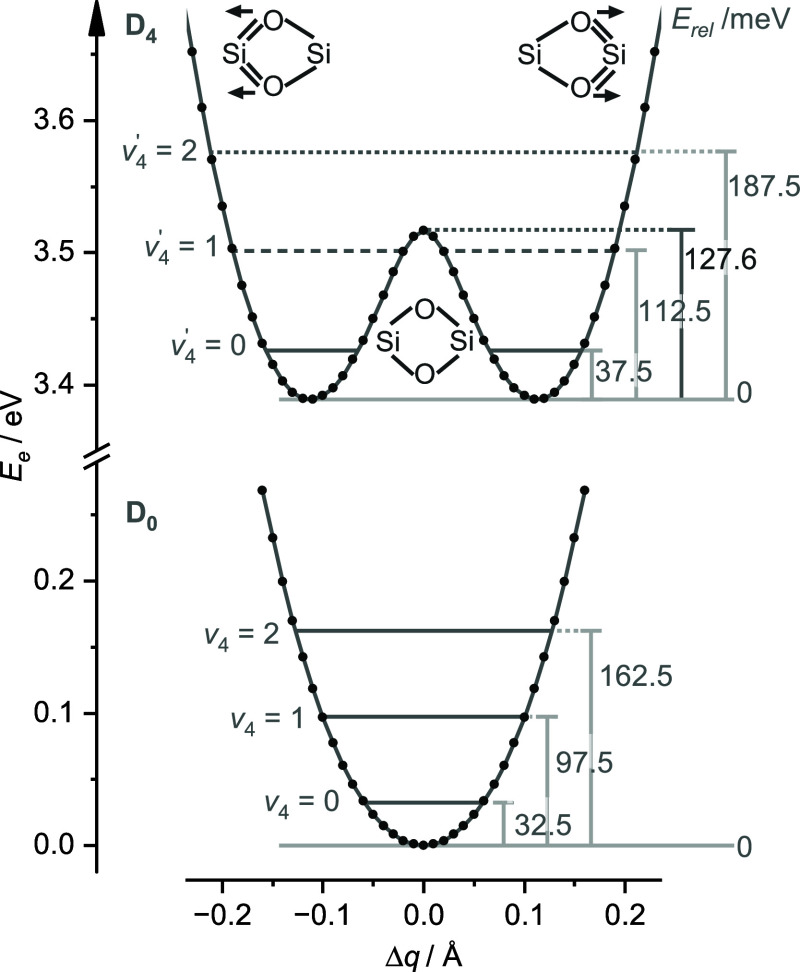
Calculated one-dimensional potential curves of the D_0_(^2^A_1_) ground and D_4_(^2^B_1_) excited states along the ν_4_ coordinate (Δ*q*) in steps of 0.01 Å obtained by single-point calculations (see text for details). Relative energies for the harmonic vibrational levels are given in meV.

In an effort to assign the vibronic structure observed for band system **B**, the D_4_ state of isomer **I** is optimized by relaxing all coordinates. This procedure results in a reduction of symmetry from *D*_2*h*_ to *C*_2*v*_ and the electronic configuration changes from ^2^B_2g_ to ^2^B_1_. The resulting energetic, structural, and vibrational data for the relaxed D_4_ state are compared in Table 3 to the corresponding data of the D_0_ ground state. The six normal modes ν_1_–ν_6_ are visualized in Figure 5 and can be classified as 2a_g_ + b_3g_ + b_1u_ + b_2u_ + b_3u_ in *D*_2*h*_ symmetry, which reduce to 3a_1_ + b_1_ + 2b_2_ in *C*_2*v*_ symmetry. Structural relaxation results in a reduction of only 831 cm^–1^ from the vertical to the adiabatic transition energy (28,455 vs 27,624 cm^–1^), indicating only a minor geometric change or a flat potential. The latter energy agrees well with the assigned band origin observed at 25,667 cm^–1^, with a discrepancy of only 1957 cm^–1^ (or 7.1%). The major geometry change is along the ν_4_ normal mode, which describes a simultaneous displacement of the two O atoms toward one of the Si atoms along the Si–Si axis. To this end, two of the Si–O bonds slightly elongate by 0.089 Å, while the other two Si–O bonds contract by 0.07 Å. As a result, the Si–Si distance changes only marginally (−0.022 Å), and the same is true for all bond angles (<4°). For symmetry reasons, the structural relaxation along the ν_4_ coordinate upon D_4_ excitation from *D*_2*h*_ to *C*_2*v*_ produces a double minimum potential, as shown in Figure 6, whereas the potential in the D_0_ state has a central single minimum along this coordinate. According to the vertical TD-DFT calculations, the transition into the D_4_(^2^B_1_) state is mainly based on the excitation 23α ← 22α (89% LUMO ← SOMO), accompanied by a small fraction of 24β ← 21β (9% LUMO+1 ← SOMO–1). The MOs and NTOs indicate that the SOMO (22) has antibonding character between all atoms. In contrast, the LUMO (23) has a stronger bonding character for the SiO_2_ bonds. Additionally, part of the transition (24 ← 21) enhances bonding between the two Si atoms. Overall, the major effect is strengthening the bonds between one of the Si atoms with the two O atoms, rationalizing the symmetry reduction in the D_4_ state from *D*_2*h*_ to *C*_2*v*_.

**Table 3:** Adiabatic Energies (eV and cm^–1^), Geometries (Å, Degree) and Vibrational Frequencies (cm^–1^) of the Optimized D_0_, D_4_ and D_5_ States of Isomer **I** of Si_2_O_2_^+^ (UB3LYP/aug-cc-pVQZ)^a^

	D_0_(^2^B_1u_)	D_4_(^2^B_1_)	D_5_(^2^B_3u_)
	*D*_2*h*_	*C*_2*v*_	*D*_2*h*_
*E*_0_	0.000	3.425 (27,624)	3.813 (30,754)
*r*_12_	1.684	1.773 (+0.089)	1.688 (+0.004)
*r*_23_	1.684	1.614 (−0.070)	1.688 (+0.004)
*r*_13_	2.409	2.387 (−0.022)	2.371 (−0.038)θ
θ_123_	91.4	89.5 (−1.9)	89.2 (−2.2)
θ_412_	88.6	85.1 (−3.5)	90.8 (+2.2)
ν_1_	869 (a_g_)	936 (a_1_, +7.7%)	824 (a_g_, −5.2%)
ν_2_	579 (a_g_)	567 (a_1_, −2.1%)	562 (a_g_, −2.9%)
ν_3_	585 (b_3g_)	536 (b_2_, −8.4%)	634 (b_3g_, −8.4%)
ν_4_	526 (b_1u_)	604 (a_1_, +14.8%)	1336 (b_1u_, +154.0%)
ν_5_	799 (b_2u_)	916 (b_2_, +14.6%)	800 (b_2u_, +0.1%)
ν_6_	308 (b_3u_)	385 (b_1_, +25.0%)	320 (b_3u_, −3.9%)

a The labeling refers to the configuration in Figure 1. The order of the vibrational modes is based on symmetry and frequency in the ground state (Figure 5). Changes of the various values compared to the D_0_ state are given in parentheses.

The one-dimensional (1D) potentials for the D_0_ and D_4_ states in Figure 6 are obtained in the following way. The ν_4_ coordinate is approximated by a translation of the two O atoms parallel to the Si–Si axis in steps of 0.01 Å, while keeping the O–O and Si–Si distances fixed at the values obtained for the optimized minimum of the D_4_(^2^B_1_) state (2.399 and 2.387 Å). Thus, the two minima in the D_4_ state correspond to the fully relaxed structures at *E*_rel_ = 0 meV and are separated by 0.22 Å. The transition state occurs at a rather low barrier of *E*_rel_ = 127.6 meV. This 1D potential is considered quite realistic, because its barrier is similar to the barrier obtained when optimizing the transition state with *D*_2*h*_ symmetry (*E*_rel_ = 116.8 meV) and an imaginary frequency of ν_4_ = *i*1012 cm^–1^. Included in the 1D potentials in Figure 6 are vibrational levels calculated for the various minima using the harmonic approximation (ν_4_ = 75 and 65 meV or 604 and 526 cm^–1^ for the D_4_ and D_0_ states). While the positions of these levels may be realistic for the single-minimum potential of the D_0_ state, they are only very rough approximations for the double-minimum potential in the D_4_ state, because the *v*_4_ = 1 level at 112.5 meV occurs very close to barrier of 127.6 meV, while higher quanta lie well above the barrier. When taking the zero-point corrected energies for the optimized minimum and transition state, the low barrier is even further reduced to 82.0 meV and thus lies below the (harmonic) *v*_4_ = 1 level. These considerations indicate that the 1D potential in the D_4_ state is rather flat along the ν_4_ coordinate and thus the harmonic approximation is quite unrealistic. Consequently, harmonic FCHT simulations for vibrational levels involving ν_4_ are not meaningful and should be taken with care. On the other hand, such simulations may still be reliable for the other vibrational modes, in particular for those exhibiting little coupling with ν_4_.

To interpret the vibronic structure of band **B**, FCHT simulations are performed for the D_4_(^2^B_1_) ← D_0_(^2^B_1u_) transition and compared to the measured EPD spectrum in Figure 4 (Table 4). For convenient comparison, all spectra are shifted such that the computed band origin aligns with the measured one (**B**_**1**_) and are plotted as a function of internal vibrational energy (i.e., by −25,667 and −27,624 cm^–1^ for panels a and b–d, respectively). Further, the simulated stick spectra are convoluted with a Gaussian line profile (fwhm = 40 cm^–1^). For clarity, only transitions with an intensity *I* > 0.001 are labeled. Initial simulations are carried out at *T* = 1 K to avoid congestion with hot band transitions. These simulations are carried out by including and excluding ν_4_ for reasons outlined above. The simulation without ν_4_ and *T* = 1 K in Figure 4b can describe already all major features rather well, with assignments of **B2** and **B3** to the expected totally symmetric ν_2_ and ν_1_ fundamentals with a_1_ symmetry. Their computed frequencies (567 and 936 cm^–1^) compare favorably with the measured values (600 and 900 cm^–1^). Following this scenario, the weaker bands **b1** and **b2** observed at 126 and −617 cm^–1^ are attributed to the hot bands 6_1_^1^ and 2_1_^0^ computed as 77 and −581 cm^–1^, respectively. FCHT simulations without ν_4_ at elevated temperatures (*T* = 300 K in Figure 4c) support this assignment. Harmonic simulations including ν_4_ and *T* = 1 K shown in Figure 4d reveal long and intense progressions of this mode, which dominate the spectrum. However, the resulting intensity pattern is not reproduced by the experiment, probably because of the anharmonic nature of the computed double-minimum potential along the ν_4_ coordinate. Nonetheless, also this simulation is consistent with the assignment of the ν_2_ and ν_1_ modes, which we thus consider quite reliable. At the current stage, the details of the rather flat D_4_ state potential remain an open question and require higher level calculations which are beyond the scope of the present work.

**Table 4:** Experimentally Obtained Frequencies and Widths (FWHM in Parentheses) Compared to the Computed Frequencies (cm^–1^) of the Franck–Condon Herzberg–Teller Simulations (Figure 5 and 7), Together with the Vibrational Assignments and Relative Intensities (*I*), for the D_4_ and D_5_ States

	Exp^a^	Calc^a^	assignment^b^	*I*^c^
Band **B**				
**B1**	0 (40)	0	0^0^	0.0299
**B2**	600 (34)	567	2^1^	0.0158
**B3**	900 (81)	936	1^1^	0.0058
**b1**	126 (96)	77	6_1_^1^	0.0046
**b2**	–617 (59)	–579	2_1_^0^	0.00017
Band **C**				
**C1**	0 (48)	0	4^1^	0.0679
**C2**	549 (49)	562	2^1^4^1^	0.0218
**C3**	809 (116)	824	1^1^4^1^	0.0029
**C4**	1106 (83)	1124	2^2^4^1^	0.0032
**C5**	1375 (146)	1386	1^1^2^1^4^1^	0.0010
**c1**	336 (144)	245	1^1^2_1_^0^4^1^	0.0017

a Relative frequencies referred to the assigned 0^0^ transitions at 25,667 and 32,216 cm^–1^(exp) and 27,624 and 32,216 cm^–1^(calc) for the D_4_ and D_5_ states, respectively.

b For hot bands, only the strongest transitions are listed.

c Only transitions with *I* > 0.001 are listed (apart from the **b2** hot band).

**Figure 7:**
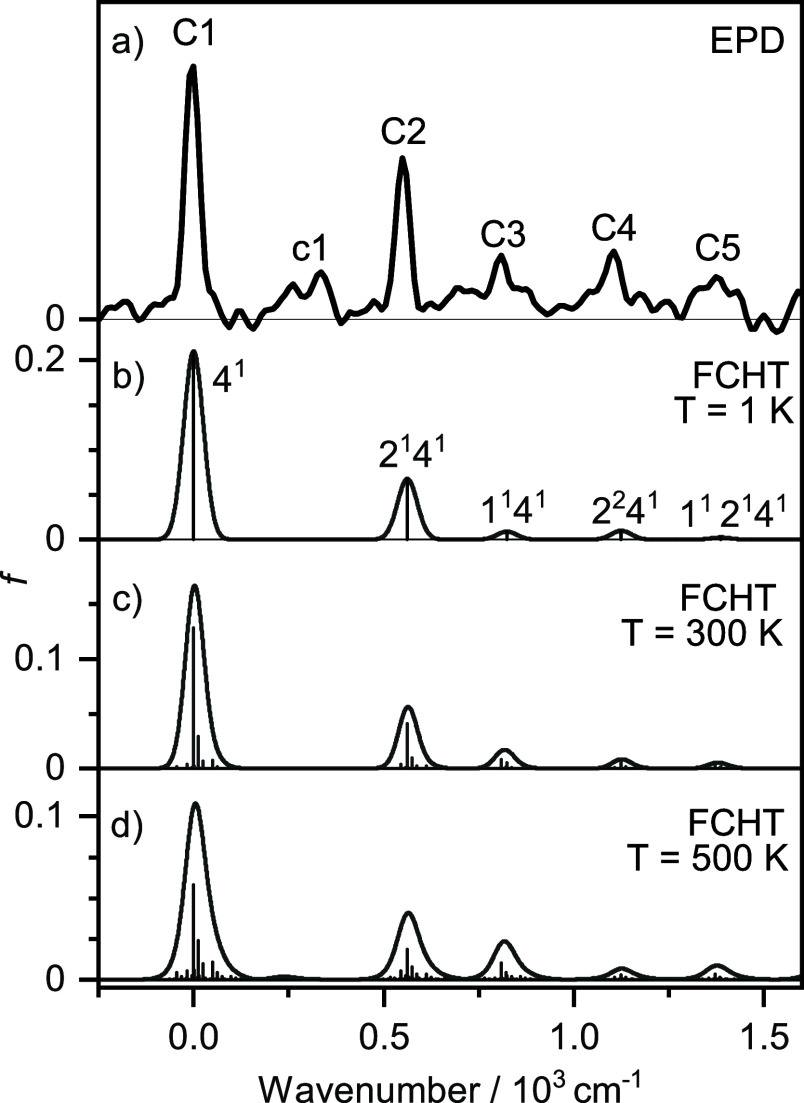
Comparison of band **C** of the experimental EPD spectrum of Si_2_O_2_^+^ (a, linear scale) to harmonic FCHT simulations (b–d, shifted by −30,754 cm^–1^) as a function of internal energy and various temperatures, along with vibrational assignments.

Band system **C** occurring in the range 32,070–33,922 cm^–1^ (294–312 nm) is characterized by five main vibronic peaks (**C1–C5**) and a weaker feature labeled **c1** (Figure 7, Table 4). The most intense peak **C1** at 32,216 cm^–1^ (fwhm 48 cm^–1^) appearing as the origin of the band system is followed by **C2** (32,765 cm^–1^, fwhm 49 cm^–1^), **C3** (33,025 cm^–1^, fwhm 116 cm^–1^), **C4** (33,322 cm^–1^, fwhm 83 cm^–1^), and **C5** (33,591 cm^–1^, fwhm 146 cm^–1^) at distances of +549, +809, +1106, and +1375 cm^–1^, respectively. The weaker feature **c1** at 32,552 cm^–1^ (fwhm 144 cm^–1^) appears at +336 cm^–1^ above **C1**. The width of the bands may again arise from a combination of the limited laser bandwidth, unresolved rotational substructure, (sequence) hot bands, and lifetime broadening. By comparison to the vertical TD-DFT calculations for isomer **I**, band system **C** is assigned to the D_5_(^2^B_3u_) ← D_0_(^2^B_1u_) transition computed at 30,560 cm^–1^ (Table 2), although its predicted oscillator strength is zero because it is dipole-forbidden in *D*_2*h*_ symmetry. As will be shown below, its intensity arises from HT coupling. According to TD-DFT, the D_5_(^2^B_1_) ← D_0_(^2^B_1u_) transition corresponds to a multicomponent excitation, primarily involving 23β ← 21β (49% LUMO ← SOMO–1), 23α ← 21α (16% LUMO ← SOMO–1), and 24α ← 22α (33% LUMO+1 ← SOMO). A small percentage originates from the excitation 22β ← 18β (3% SOMO ← SOMO–4). In addition, we note that the TD-DFT calculations yield strong spin contamination of the D_5_(^2^B_3u_) doublet state, which may indicate mixing with the nearby Q_0_(^4^B_3u_) quartet ground state separated by a few meV (0.6 or 25 meV from the vertical and adiabatic D_5_ energy, respectively).

To assign the vibronic structure of band system **C**, the D_5_ state is optimized. In contrast to the D_4_ state, geometry relaxation of the D_5_ state does not reduce the symmetry from *D*_2*h*_. The resulting energetic, structural, and vibrational parameters are listed in Table 3. The geometry change upon D_5_ excitation is rather small, and as a result, the adiabatic and vertical excitation energies are rather similar (30,754 and 30,560 cm^–1^). The small geometry change upon D_5_ excitation is consistent with the intense origin band and the sparse vibronic activity of band **B**. As mentioned before, the D_5_(^2^B_3u_) ← D_0_(^2^B_1u_) transition is dipole-forbidden in *D*_2*h*_ symmetry but may gain intensity via HT coupling induced, for example, by a vibrational mode with b_1u_ symmetry.^97−99^ Indeed, structural elongation of isomer **I** of Si_2_O_2_^+^ along the ν_4_(b_1u_) mode reduces the symmetry from *D*_2*h*_ to *C*_2*v*_ (much like in the D_4_ state) and transforms the forbidden ^2^B_3u_ ← ^2^B_1u_ transition into an allowed ^2^B_1_ ← ^2^A_1_ transition. Similar to the geometry, the vibrational frequencies do not change much upon D_5_ excitation, apart from ν_4_ whose frequency increases by a factor 2.5.

In a next step, the vibronic structure of the forbidden ^2^B_3u_ ← ^2^B_1u_ transition activated by HT coupling with the ν_4_(b_1u_) mode is predicted by FCHT simulations and the resulting spectra obtained for various temperatures (*T* = 1, 300, and 500 K) are compared in Figure 7 to the measured EPD spectrum (Table 4). To facilitate comparison with the measured spectrum, the computed stick spectra are convoluted with Gaussian line profiles with fwhm = 40 cm^–1^. Because of HT coupling, the ν_4_ fundamental acts in the simulations as a false origin and is assigned to the experimental band origin (**C1**), and the simulated spectrum has been shifted accordingly to match ν_4_ with **C1** (by −32,216 and −30,754 cm^–1^ for panels a and b-d). Indeed, very good agreement is observed between the FCHT simulations and the measured EPD spectrum with respect to both the relative band positions and intensities, with deviations of ≤18 cm^–1^ for **C2–C5**, which is well below the observed widths of the bands. According to the FCHT selection rules, the vibrational combination and overtone bands starting at the ν_4_ fundamental (4^1^) acting as false origin must be totally symmetric and thus of a_g_ symmetry in *D*_2*h*_. This includes the ν_1_ and ν_2_ fundamentals and their progressions and combinations, as well as even quanta of the other modes. Indeed, as indicated in Figure 7 and Table 4, the bands **C2–C5** can readily be assigned to 4^1^2^1^, 4^1^1^1^, 4^1^2^2^, and 4^1^1^1^2^1^, respectively. The experimental values derived for ν_1_ = 809 cm^–1^ and ν_2_ = 549 cm^–1^ are in good agreement with the values of ν_1_ = 824 cm^–1^ and ν_2_ = 562 cm^–1^ computed for the D_5_ excited state. The FCHT simulations at elevated temperatures suggest that band **c1** arises from a variety of hot bands, with 4^1^1^1^2_1_ being the dominant one. This assignment provides a rough estimate of ν_2_ = 809–336 = 473 cm^–1^ in the D_0_ state, consistent with the predicted value of 579 cm^–1^, when taking into account the limited spectral resolution.

## 4 Conclusions

In summary, the geometric, vibrational, and electronic structures of the Si_2_O_2_^+^ cation are characterized by EPD spectroscopy in a tandem mass spectrometer coupled to a laser vaporization source and complementary TD-DFT calculations at the UB3LYP-D3/cc-pVQZ level coupled with FCHT simulations. Significantly, these experimental and computational data correspond to the first spectroscopic data for the optical and photochemical properties of this simple tetra-atomic silicon oxide cation, which may be a reaction intermediate in the formation of the silica and silicate dust grains abundant in the ISM. As such, the new data provide valuable input for astrochemical models describing the formation of such grains in a bottom-up approach via an ionic route at the molecular level. Due to its high dissociation energy of around 4.6 eV, Si_2_O_2_^+^ may survive the harsh conditions in the high temperature regions of the ISM and our laboratory spectra may facilitate its astronomical detection.

The EPD spectrum reveals three electronic transitions **A–C** near 519, 390, and 310 nm, which have been assigned to the transitions from the D_0_(^2^B_1u_) ground state of the most stable planar rhombic isomer **I** with *D*_2*h*_ symmetry into the D_1_(^2^A_g_), D_4_(^2^B_2g_), and D_5_(^2^B_3u_) excited states. The EPD spectrum is observed in the lowest-energy dissociation channel composed of the rather stable SiO and SiO^+^ fragments, which requires the resonant absorption of at least two photons. Band **A** occurs close to half of this two-photon threshold, which may explain its appearance as weak and unresolved transition in the EPD spectrum, although the computations predicted the assigned D_1_ state as the most intense dipole-allowed state by far in the range below 5 eV. The other two transitions **B** and **C** display resolved vibronic structure, which has been assigned to vibrational activity in the assigned D_4_ and D_5_ excited states with band origins at 25,667 and 32,216 cm^–1^, respectively. According to TD-DFT, the optically allowed D_4_ state undergoes symmetry reduction from *D*_2*h*_ to *C*_2*v*_ along the ν_4_(b_1u_) coordinate upon geometry optimization, resulting in a double minimum potential with a low barrier at the *D*_2*h*_-symmetric transition state. This prediction complicates the vibronic assignment and should be verified in future studies by higher-level calculations. Nonetheless, the analysis provides reliable frequencies for the two totally symmetric modes in the excited D_4_ state (ν_1_ = 900 cm^–1^, ν_2_ = 600 cm^–1^). In contrast to the D_1_ and D_4_ states, the assigned D_5_ state is dipole-forbidden and gains its intensity by HT coupling involving the ν_4_(b_1u_) mode. Hence, the band origin observed at 32,216 cm^–1^ in the EPD spectrum corresponds to a false origin and represents the ν_4_ fundamental in the D_5_ state, which then also occurs in combination with the totally symmetric modes (ν_1_ = 809 cm^–1^, ν_2_ = 549 cm^–1^).

In addition to our recent studies on Si_2_^+^, Si_2_O^+^, SiO_2_^+^, and Si_3_O_2_^+^, the combined spectroscopic and computational approach applied herein to Si_2_O_2_^+^ has proven a successful strategy for revealing the first optical and photochemical properties of small polyatomic Si_*n*_O_*m*_^+^ cations. All of these small Si_*n*_O_*m*_^+^ cations produce SiO in their lowest-energy photodissociation channels, which thus may contribute to the high abundance of this molecule in the ISM. Although comparison of the vibronically resolved EPD spectrum with the astronomical spectrum of the diffuse interstellar bands (DIBs)^100−103^ is complicated by the various broadening mechanisms and the different nature of the physical processes involved (two-photon EPD versus single-photon absorption), no obvious match is suggested. In general, our EPD spectra of these small Si_*n*_O_*m*_^+^ cations provide valuable benchmarks for developing and testing accurate computational approaches for accurately calculating higher excited states of these open-shell radical cations, which still is rather challenging. Future efforts using the same spectroscopic and computational approach include larger Si_*n*_O_*m*_^+^ cations, metal-containing silicates,^29,33,34^ and Si_*n*_C_*m*_^+^ cations.^73^ Experimentally, we will implement a cryogenic ion trap into the setup to fully thermalize the ions to low temperatures (similar to conditions in the ISM), because the plasma expansion of the laser vaporization source typically results in temperatures ranging from 300 to 1000 K leading to substantial spectral congestion arising from hot bands.^74^
